# Characterization of the OmyY1 Region on the Rainbow Trout Y Chromosome

**DOI:** 10.1155/2013/261730

**Published:** 2013-03-11

**Authors:** Ruth B. Phillips, Jenefer J. DeKoning, Joseph P. Brunelli, Joshua J. Faber-Hammond, John D. Hansen, Kris A. Christensen, Suzy C. P. Renn, Gary H. Thorgaard

**Affiliations:** ^1^School of Biological Sciences, Washington State University Vancouver, 14204 NE, Salmon Creek Avenue, Vancouver, WA 98686-9600, USA; ^2^Center for Reproductive Biology, Washington State University, Pullman, WA 99164-7520, USA; ^3^School of Biological Sciences, Washington State University, Pullman, WA 99164-4236, USA; ^4^US Geological Survey, Western Fisheries Research Center, 6505 NE 65th Street, Seattle, WA 98115, USA; ^5^Biology Department, Reed College, 3203 SE Woodstock Boulevard, Portland, OR 97202-8199, USA

## Abstract

We characterized the male-specific region on the Y chromosome of rainbow trout, which contains both sdY (the sex-determining gene) and the male-specific genetic marker, OmyY1. Several clones containing the OmyY1 marker were screened from a BAC library from a YY clonal line and found to be part of an 800 kb BAC contig. Using fluorescence *in situ* hybridization (FISH), these clones were localized to the end of the short arm of the Y chromosome in rainbow trout, with an additional signal on the end of the X chromosome in many cells. We sequenced a minimum tiling path of these clones using Illumina and 454 pyrosequencing. The region is rich in transposons and rDNA, but also appears to contain several single-copy protein-coding genes. Most of these genes are also found on the X chromosome; and in several cases sex-specific SNPs in these genes were identified between the male (YY) and female (XX) homozygous clonal lines. Additional genes were identified by hybridization of the BACs to the cGRASP salmonid 4x44K oligo microarray. By BLASTn evaluations using hypothetical transcripts of OmyY1-linked candidate genes as query against several EST databases, we conclude at least 12 of these candidate genes are likely functional, and expressed.

## 1. Introduction

Salmonid fishes have the XX/XY system of sex determination [1]. Most rainbow trout have morphologically distinguishable sex chromosomes [2], with the short arm being longer in the X chromosome than the Y, primarily due to presence of 5S rDNA sequences on the X chromosome (reviewed in [3]). Some wild fish and several male clonal lines, including the Swanson YY and Arlee YY clones have a Y chromosome that resembles the X chromosome in having a longer short arm including the 5S rDNA sequences [4]. This fact and the viability seen in rainbow trout and Chinook salmon YY individuals [5, 6] suggest that the X and Y chromosomes share considerable genetic content.

 The SEX locus is not found on a common linkage group in Pacific salmon and trout, but rather each species has the SEX locus on a different linkage group as shown by genetic mapping and localization of male-specific markers using in situ hybridization with clones to GH-Y [7–9]. (Rainbow and cutthroat trout are an exception to this [10], and hybrids between these two species are interfertile.) Although it is possible that each species has a different master sex determining gene, this is unlikely because there are several male-specific markers shared by the *Oncorhynchus *species. These include OmyY1, found in all Pacific trout and salmon [11], OtY2 [12] which is related to OmyY1, and GH-Y [13], which are found in most species of Pacific salmon, and OtY1, found only in Chinook salmon [14]. These results support the hypothesis that a small chromosomal segment containing the SEX locus, or the small short arm containing the SEX locus, is transposing to a new chromosome in each of these species (reviewed in [9, 15]).

The OmyY1 genetic marker, which has been used to sex fish from all of the *Oncorhynchus* species, was isolated from lambda libraries prepared from male fish of both rainbow trout (OSU × Hotcreek) and Chinook salmon (where the region is referred to as OtY3) [11]. Recently, evidence has been presented supporting the hypothesis that an immune function related-gene, sdY, which is found within the rainbow trout OmyY1 lambda clone, may be the master sex determining gene for many species in the Salmoninae [16]. We present evidence based on sequence information of BAC clones isolated from the OmyY1 region of rainbow trout that there are a number of sex-linked genes shared between the X and Y chromosomes, implying the sex chromosomes are in the early stages of differentiation. Some of the genes in the region adjacent to sdY show male-specific expression. 

## 2. Materials and Methods

### 2.1. Doubled Haploid Rainbow Trout Clonal Lines and Crosses

Type I loci and other markers were sequenced in four doubled haploid parental lines produced by androgenesis (female Oregon State University (OSU), female Whale Rock (WR), male Swanson (SW), and male Arlee (AR)). Markers were mapped in a cross of (OSU × AR) to determine concordance with phenotypic sex. These fish were previously used to generate a dense genome-wide linkage map [17–19] to analyze quantitative trait loci for a variety of traits [20, 21] and to characterize linkage of markers on the Y chromosome [4, 22]. In all crosses, sex phenotype was determined by internal examination of gonads.

### 2.2. BAC Library Screening for OmyY1 Marker Associated BACs

High density filters corresponding to the 5X BAC DH YY male Swanson (SW) library (EcoR1 set) for rainbow trout were screened using [^32^P] dCTP-labeled amplicons from the OmyY1 region. For screening the filters, two probes were generated by PCR amplification of SW genomic DNA using previously described primers to the OmyY1 region [11]. These amplicons were TOPO TA cloned and transformed in JM107 *E. coli*. After identification and isolation of OmyY1+clones, plasmid DNA was extracted and used as a probe to screen the filters. The product was purified (Qiagen) and then labeled with ^32^P-dCTP (Amersham) using Ready-to-Go beads (GE Healthcare) according to the manufacturer's suggestions. The labeled probe was purified using G-50 columns (ProbeQuant, GE Healthcare) and then used for hybridization. Filters were hybridized (5X SSC, 1% SDS, 0.5% sodium pyrophosphate, 0.5% nonfat milk and 10% dextran sulfate at 65°C) and then washed (0.5% SSC/0.5% SDS, 65°C) under stringent conditions. Positive clones were confirmed by PCR using primer sets corresponding to OmyY1. This resulted in many hits, but only the strongest signals were further evaluated by PCR and FISH. BACs were named according to the coordinates on the original plate. Clones were obtained from the National Center for Cool and Cold Water Aquaculture, ARS-USDA as stab cultures. BAC DNA for PCR confirmation was prepared from selected clones grown in 300 mls LB broth + chloramphenicol (12.5 *μ*g/mL) overnight at 37C with shaking at 250 rpm, and DNA was isolated using a Qiagen Plasmid Maxi Kit.

### 2.3. PCR Screening of OmyY1 Marker Associated BACs

Further PCR screening of the selected clones was performed in a PTC100 or 200 Thermalcycler (MJ Research) using primers noted above. Reactions were carried out in 10 *μ*L volumes containing 0.5 U Taq DNA Polymerase (GenScript), .4 uM primers, 1 *μ*L 10x PCR buffer containing 1.5 mM MgCl_2_, 200 *μ*M dNTPs, and 1 *μ*L of 40 ng BAC DNA template. PCR conditions comprised of an initial denaturation step of 95°C for 5 min; 35 cycles with a denaturation step of 95°C for 30 sec, 45 sec at specific annealing temperature, and extension at 72°C for 45 sec followed by a final extension at 72°C for 5 minutes. PCR products were separated on a 1.2% agarose gel containing .5x TBE and GelRed Nucleic Acid stain (Phenix). The DNA fragments were visualized using a Gel Logic 100 system and UV trans-illuminator (Fisher Biotech).

### 2.4. Assembly of the OmyY1 BAC Contig

BAC clones RE223F6 and RE143K8 were confirmed using OmyY1-specific PCR primers and sent to Ming Chen Luo at University of California, Davis for assembly of a contig with the Swanson (SW) physical map [23]. Contig #6256 was identified as encompassing the OmyY1 BACs and included 60 additional BACs. Eleven BACs spanning the contig were ordered to use for FISH and end-sequencing and included RT399M07, RT278E18, RT423A24, RT399C14, RT439A05, RT578J04, RT450D22, RT015B10, RT492A21, RT004L12, and RT225N07. After BAC DNA isolation as described above, and determination of DNA concentration, aliquots were sent to the WSU Pullman Molecular Biology Core for end-sequencing using either T7, SP6, or M13 primers.

### 2.5. Localization of OmyY1 BAC Clones on Rainbow Trout Chromosomes

Blood was cultured from rainbow trout using standard methods [4]. Rainbow trout BAC DNA was labeled with Spectrum Orange using a nick translation kit (Abbott Molecular). Human placental DNA (2 *μ*gs) and Cot-1 DNA (1 *μ*g, prepared from rainbow trout) were added to the probe mixture for blocking. Hybridizations were carried out at 37°C overnight and posthybridization washes were as recommended by the manufacturer (Abbott Molecular) with minor modifications [24]. Antibodies to Spectrum Orange (Molecular Probes) were used to amplify the signal. Slides were counterstained with 4,6-diamidino-2-phenylindole (DAPI) at a concentration of 125 ng DAPI in 1 mL antifade solution (Abbott Molecular). Images were captured with a Jai camera and analyzed with Cytovision Genus (Applied Imaging, Inc.) software. Chromosomes were arranged according to size within the metacentric/submetacentric and acrocentric groups.

### 2.6. Characterization of the OmyY1 BAC Contig: Illumina Sequencing

A minimum tiling path of BAC clones from the OmyY1 region was sequenced by Amplicon Express, Inc. Pullman, WA by Focused Genome Sequencing (FGS). FGS is a next-generation sequencing (NGS) method developed at Amplicon Express that allows very high quality assembly of BAC clone sequence data using the Illumina HiSeq (San Diego, CA). The proprietary FGS process makes NGS tagged libraries of BAC clones and generates a consensus sequence of the BAC clones. 

Five OmyY1 BACs spanning the OmyY1 physical map contig (RT578J04, RT450D22, RT015B10, including BAC clones RE223F06, and RE143K08 containing the OmyY1 locus) were sequenced using Roche 454 GS FLX next-generation sequencing technologies, by the WSU Pullman bioinformatics genomics core lab. This sequence evaluation yielded 94325 total reads, assembling 188 contigs ranging in size from 50.4 kb to <100 bp.

### 2.7. Characterization of the OmyY1 BAC Contig: Comparative Genomic Hybridization

We used a 4 x 44 k Agilent salmonid microarray [25] to determine gene homologies within the OmyY1 contig through Comparative Genomic Hybridization (CGH). This microarray was designed using 60 bp oligos from the 3′ end of most known cDNAs from Rainbow Trout or Atlantic salmon. We pooled six clones from the contig (RT492A21, RT450D22, RT015B10, RT423A24, RT004L12, and RE143K08) and labeled with both Alexa Fluor 5 and and Alexa Fluor 3 using the BioPrime Total Genomic Labeling System (Invitrogen). Samples were hybridized to the salmonid microarray in duplicate versus unrelated BAC clones using the manufacturer's protocol for CGH (Agilent). Using the R statistical software package, microarray data were floored at 2 standard deviations above the local background then normalized with a LOESS normalization method. Color ratios were fit to a Linear Model and then analyzed using empirical Bayes statistics [26]. Positive hits on the array features with significant *P* values and higher fluorescence intensity for OmyY1 contig samples than the competing samples from unrelated BAC clones.

False positives were removed from the initial list of positive hits by conducting a stand-alone BLAST of the 60 bp oligo sequences from the array against a first draft rainbow trout genome (M. R. Miller, unpublished) and removing any repetitive elements. A second BLAST inquiry was conducted in GenBank, and additional repetitive elements were removed from the list. Additionally, vector (*E. coli*) genomic DNA was hybridized to the microarray, and overlapping hits with those from the OmyY1 contig positive hit list were removed. The final positive hit list represented possible candidates for functional genes closely linked to OmyY1. Genes of interest were confirmed by searching the Illumina assembly and/or by PCR using primers designed to the EST target in the OmyY1 BAC clones and rainbow trout genomic DNA, followed by Sanger sequencing on an ABI 3130xl.

Once possible candidates for functional genes were confirmed within the OmyY1 BAC contig, the corresponding BAC sequences were compared to EST sequences from the 4 x 44 k salmonid microarray annotation file, the cGRASP rainbow trout EST database (http://web.uvic.ca/grasp/), or cDNA sequences from GenBank. In addition, the list of possible functional genes was BLAST-analyzed against both the rainbow trout and Atlantic salmon [27] draft genome assemblies to identify potential paralogs and eliminate additional repetitive sequences. Candidate genes were proposed to be functional within the OmyY1 contig if a full-length hypothetical mRNA could be identified within the BAC contig sequence. Criteria for functionality included presence of start and stop codons, with no in-frame stop codons. Gene identity required corresponding BAC sequence to be 100% identical to candidate genes, excluding compressions found in 454 and Illumina sequencing of homopolymers. For microarray hits not found in the BAC contig assembly, corresponding rainbow trout-specific expressed sequences were compared to Sanger sequenced PCR products from OmyY1 contig BAC clones or to OmyY1-linked scaffolds from the draft rainbow trout genome assembly.

### 2.8. PCR Confirmation and Sanger Sequencing of BAC Clones Containing Loci Identified in BLAST Analysis of Illumina Sequencing Results and SNP Identification

GenBank BLASTn and BLASTx analyses were performed on contig sequences obtained from the Illumina/454 OMY BACs assembly of the 6 OmyY1 BAC clones spanning the minimum tiling path of the physical map contig #6256. Following identification of regions of homology within contigs to various teleost annotated gene loci, the resulting sequences were subsequently screened for suspected and known repetitive elements in salmonids using a salmonid-specific repeat masker (http://lucy.ceh.uvic.ca/repeatmasker/cbr_repeatmasker.py).

PCR primers were designed either to the OMY BAC contig sequence itself, or to the GB cDNA or EST sequence available (see Table 3). Additionally, subsequent to the Comparative Genomic Hybridization (CGH) results, ESTs identified as positive hits were also used for template in target design. ESTs identified by CGH were aligned with the rainbow trout draft genome sequence, and scaffolds with significant homology provided another source for primer design.

PCR amplified products were treated with 2 U of FastAP (Fermentas) and 1 U Exonuclease I (USB) and sequenced directly using 2.3 uls of the sequencing RR mix from the Big Dye Terminator v3.1 cycle sequencing kit (ABI), 2 *μ*ls of the provided 5x buffer, .4 *μ*m primer, and .5–2 *μ*L of the PCR reaction. Reactions were run for 29 cycles with 4 minute extension at 72C, then cleaned by CleanSEQ magnetic bead separation (Agencourt) and run on an ABI 3130xl sequencer. Sequence reads were aligned using DNASTAR Seqman II v5 sequence analysis software.

## 3. Results

### 3.1. The OmyY1 BAC Contig

**Figure 1:**
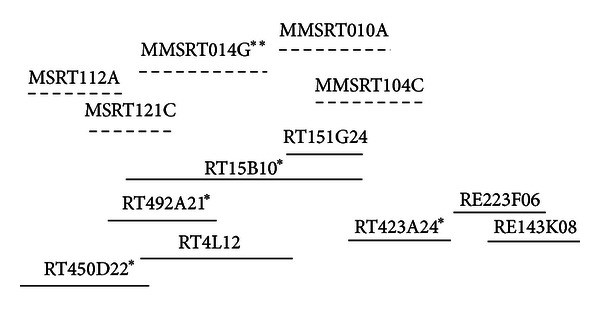
Diagram of the OmyY1 BAC contig showing clones that we examined in this project. The dashed lines above the BAC clones indicate Scaffolds from Mike Miller's rainbow trout genome assembly. *Indicates that the clone was sequenced by Illumina.**Indicates scaffolds that did not include BACs known to be in the OmyY1 contig (6256), but which apparently had BACs that are in this region because sequences of genes identified in the OmyY1 BACs matched the sequence of genes in these scaffolds exactly.

Three BAC clones containing the OmyY1 sequence were confirmed following screening of the Swanson rainbow trout YY RE library. These clones overlapped with a contig from the Swanson RT library which originally contained 30 clones. Most recently a third generation BAC physical map has been prepared with BACs from the three Swanson BAC libraries: RE (EcoRI), RB (BamHI), and RT (HindIII) (Y. Palti, pers. com.). This updated map expands the previous OmyY1 BAC-containing contig, now contains 60 clones, and is designated as #6256. Although a PCR product was obtained following amplification with OmyY1 primers in most of the clones, we were able to sequence the OmyY1 product from only two of the RE BAC clones. The proposed sex determining gene, sdY [16], is found in the same two RE BAC clones and is contained within the 22 kb OmyY1 GenBank deposition. A diagram showing the clones from physical map contig #6256 analyzed in this project is shown in Figure 1.

### 3.2. Localization of OmyY1 BAC Clones on Rainbow Trout Chromosomes

**Figure 2:**
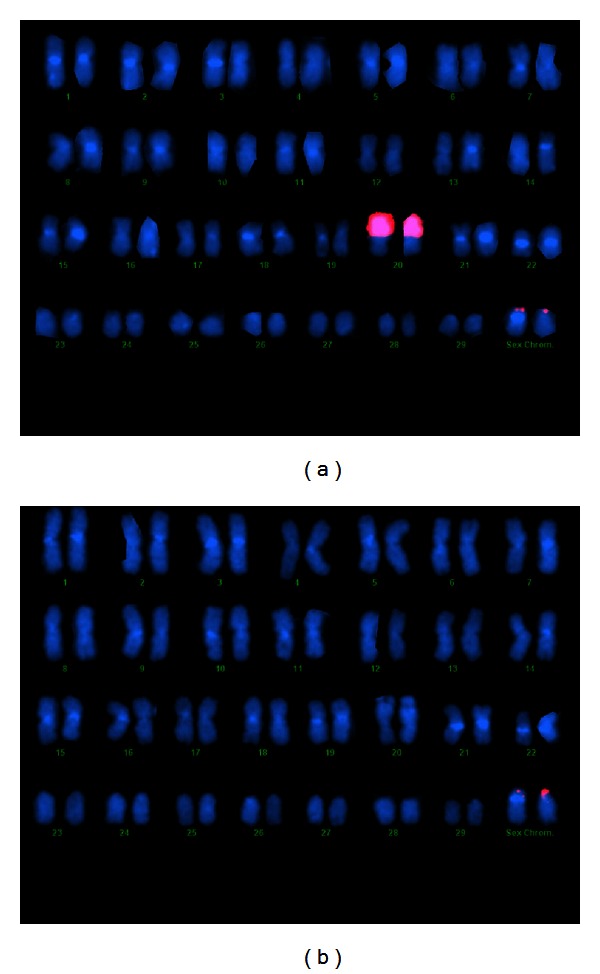
(a) Hybridization of the BAC clone RE143K08 (which is found near the 3′ end of the OmyY1 contig and contains the OmyY1 marker, the sdY gene and ribosomal DNA) to male rainbow trout chromosomes. Note the signals on the sex chromosome pair and chromosome pair 20, the site of the 18S rDNA locus in rainbow trout. (b) Hybridization of the BAC clone RT429A21 to male rainbow trout chromosomes. This BAC is close to the 5′ end of the OmyY1 contig (6256) (see Figure 1). Note the larger signal on the Y chromosome compared to the X.

A dozen BAC clones from physical map contig #6256 were used as probes in FISH (fluorescence *in situ*) experiments for hybridization to rainbow trout chromosomes. All of these clones hybridized to the end of the short arm of the Y chromosome of rainbow trout with some signal observed on the end of the short arm of the X chromosome in many cells (Figures 2(a) and 2(b)). Hybridization was done on chromosomes prepared from OSU × Hot Creek hybrids. The Hot Creek strain has a Y chromosome which is morphologically distinct from the X, so the X and Y chromosomes can be easily distinguished, with the short arm of the Y being smaller than the short arm of the X chromosome [2]. Some of the BAC clones contained ribosomal DNA and those clones also hybridized to the NORs on chromosome 20 in rainbow trout (Figure 2(a)). BAC clones in the middle of the contig hybridized only to the Y chromosome in many cells (Figure 2(b)). These clones did not contain ribosomal DNA. The BAC clones containing OmyY1 and sdY did not hybridize to the X or Y chromosomes in Chinook Salmon (data not shown). As mentioned above, these clones contained 18S ribosomal DNA and hybridized only to known sites of rDNA on Chinook chromosomes. 

### 3.3. Characterization of the OmyY1 BAC Contig: Illumina and 454 Sequencing Results

Five BACs spanning the OmyY1 physical map contig #6256 (RT450D22, RT015B10, RT492A21, RT004L12, and RT423A24) were Illumina sequenced and assembled by Amplicon Express (http://www.ampliconexpress.com/). The BAC clones were sequenced by Focused Genome Sequencing (FGS). FGS is a next-generation sequencing (NGS) method developed at Amplicon Express that allows very high quality assembly of BAC clone sequence data using the Illumina HiSeq (San Diego, CA). The proprietary FGS process makes NGS tagged libraries of BAC clones and generates a consensus sequence of the BAC clones. Illumina sequencing yielded an average of 2,412,973 reads per BAC clone assembling into 56 contigs ranging in size from approximately 245 kb to 1 kb. Additionally, five OmyY1 BACs spanning the OmyY1 physical map contig (RT578J04, RT450D22, RT015B10, including BAC clones RE223F06, and RE143K08 containing the OmyY1 locus) were sequenced using Roche 454 GS FLX next-generation sequencing technologies, by the WSU Pullman bioinformatics genomics core lab.

Assembled DNA sequence contigs were evaluated directly for gene coding content using NCBI-BLASTx [28] and also redundantly masked for repetitive elements using the salmonid-specific repeat masker prior to NCBI-BLASTx evaluation, to identify candidate gene sequences. BLASTx coding content homologies provided access to GenBank gene sequence data depositions, which were then used in NCBI distributed stand-alone BLAST-2.2.25 application evaluations. The contigs containing the OmyY1 BAC clone illumine sequence data were put into the Standalone BLAST databases format and then BLASTn was conducted on this OmyY1 database with downloaded candidate gene sequences for comprehensive gene sequence content homology ([16, 29] and http://web.uvic.ca/grasp/). When assembled, sequence contigs were found to sequentially contain gene coding-region content, and these adjacent assemblies were evaluated for flanking DNA sequence homology to allow assembly of larger continuous sequence reads spanning the candidate gene sequence. Candidate gene sequences were characterized for intron-exon boundaries by sequence alignment with GenBank mRNA or predicted gene sequence depositions.

By this method the following 12 predicted protein coding gene sequences have been identified (including sdY), partially annotated and deposited to GenBank (Table 1). Five of these genes have confirmed transcripts in at least one of five different databases that were searched and these are reported in Table 2. Additionally, one predicted pseudogene was identified in the contig #6256 sequence assemblies (Table 1), determined by presence of premature stop codons relative to annotated predicted proteins from other species (data not shown). Further BLAST analyses within the cGRASP EST cluster rainbow trout databases yielded an additional 5 OmyY1 linked transcripts for unknown genes: omyk-BX860091, omyk-CB488087, Contig22207, omyk-DV199273, and Contig19333 (Tables 1 and 2). BLASTx in GenBank revealed that omyk-BX860091 shows poor homology to laminin subunit beta-1-like (best homology is 63% identity to *Oreochromis niloticus*) and Contig22207 has partial homology to Sec16A (all homology <55% identity for <25% of the transcript). No predicted protein sequences for these unknown genes could be analyzed.

### 3.4. Characterization of the OmyY1 BAC Contig: Comparative Genomic Hybridization Results

A 4 x 44 k Agilent salmonid microarray [25] was used to identify gene homologies within the OmyY1 contig. The preliminary list of hits from the OmyY1 contig contained 124 expressed sequences. Following the removal of duplicates within the array and false positive hits by removing overlapping hits with unrelated BAC clones, we were left with 48 ESTs (see Supplemental File 1 of the Supplementary Material available online at http://dx.doi.org/10.1155/2013/261730). This list was compared to genes found in the Illumina and 454 assemblies to confirm that the CGH yielded true positives, with six genes found on both lists: cAMP-responsive element-binding protein 3-like protein 4, F-Box/WD repeat containing protein 2, Na/K/2Cl co-transporter, CREB-regulated transcription coactivator 2, DENN domain-containing protein 4B, and Zinc transporter ZIP1. No other genes confirmed in the OmyY1 contig assemblies were represented on the 4 x 44 k oligo array by exact name or alias. 

Stand-alone BLAST analysis was conducted with the 48 array hits using a draft *Oncorhynchus mykiss* genome assembly as the database. This draft genome assembly included sequence from 7 BAC clones within the rainbow trout physical map contig #6256; 5 of which were chosen for Illumina and 454 sequencing in our study (Figure 1). Microarray sequences matching any corresponding assembled genome scaffolds were confirmed as OmyY1-linked. Of these 7, scaffolds MMSRT112A (containing sequence from RT450D22) and MMSRT121C (containing sequence from RT492A21) showed significant sequence homology and coverage to 5 microarray hits each, 3 of which fall in an overlapping region between both BAC RT450D22 and BAC RT492A21clones (supplemental file 1). 

These microarray results identified additional sex-linked or paralogous scaffolds that contained a significant number of microarray hits. Stand-alone BLAST results showed MMSRT069D had significant homology to 4 hits (Supplemental File 1). To check whether this scaffold was sex-linked or paralogous, primers were designed to the microarray EST sequences then partial gene sequences were amplified from the OmyY1 BAC clones and sequenced. Sequences from MMSRT069D showed 90% homology to OmyY1 BAC sequence indicating the scaffold was most likely a paralogous scaffold, amplifying in BAC clone RT15B10. There were also five rDNA and/or repetitive ESTs that contained homology to similar sets of draft genome scaffolds, including MMSRT092E, 100C, 059D, 036A, 103G, 136F, and 084G (Supplemental File 1). The 5 EST sequences had similar rates of homology with both the draft genome and OmyY1 contig assemblies, but none show with 100% homology with any assembly. Due to the difficulty in amplifying clean rDNA and repetitive sequences in the BACs, and because a great number of scaffolds showed identical homology to the ESTs, we could not determine whether these scaffolds were paralogous or identical to the OmyY1 region. It is worth noting, however, that MMSRT100C also contains IRF9 (interferon regulatory factor 9) which is the gene from which *sdY* was originally derived [16]. This suggests that even though these rDNA and repetitive sequences are found in many locations throughout the genome, there are specific sex-linked versions surrounding OmyY1 and sdY. 

The 48 CGH hits queried in stand-alone BLAST evaluations of the OmyY1 contig sequence and draft genome assemblies were checked against GenBank (using BLASTn and BLASTx) for homology with known salmon and trout repetitive elements and checked against the rainbow trout draft genome. This search showed 17 hits represented transposable elements, 5 contained ribosomal DNA, and 12 more were repetitive, leaving 14 ESTs showing significant nonrepetitive sequence homology to the OmyY1 region (Supplemental file 1). Although repetitive hits were not included in Tables 1 and 2, many of these repetitive ESTs from the microarray seem to be expressed within the OmyY1 contig and may still be functionally and evolutionarily important (details of both BLAST searches are included in Supplemental File 1). It is also important to note that for some ESTs, repetitive elements only made up a small portion of the entire transcript.

 Some ESTs within the 4 x 44 k annotation file were derived from Atlantic Salmon (*Salmo salar*), and for those ESTs with less than 99-100% homology to either the *O. mykiss* draft genome assembly or the OmyY1 contig Illumina assembly, rainbow trout-specific EST databases were searched to find expressed orthologs or paralogs. These databases include the cGRASP *O. mykiss* EST cluster databases, GenBank, Salem's 454 database [29], and the *O. mykiss* sex-specific gonad EST databases at 35dpf from Yano et al. [16]. Found orthologs and paralogs were evaluated for strong homology and comprehensive gene coverage within OmyY1 contig sequence and for the lack of premature stop codons when predicted protein sequences were available to determine functionality of genes. 

 The final list of predicted functional, nonrepetitive genes within the OmyY1 contig contained 11 hits from the 4 x 44 k microarray (Table 2). Six of these ESTs (cr3l4, FBXW2, nkcc1a, TORC2-like, and ZIP1 (with 2 predicted splice variants)) were found in the Illumina assembly for the OmyY1 contig. 

### 3.5. Analysis of SNPs from the OmyY1 BACs

For further confirmation of these loci within the BAC clones, Sanger sequencing was performed. PCR primers were designed either to the OMY BAC Illumina/454 contig sequence, GB cDNA, or EST sequence available, or to the *O. mykiss* draft genome homologous scaffolds (see Figure 1). The F-box and WD repeat domain containing 2 (Fbxw2) gene comprises a 7.6 kb region within BAC RT450D22. It was evaluated for sequence polymorphisms in the DH RT clonal lines AR, OSU, SW, and WR, [30, 31] yielding 10 SNPs; 4 of which were population specific and 6 of which appeared sex specific. All SNPs are referenced against the Fbx numbering system of the GenBank deposition and locations are tabulated (see Table 3). 

 Gene-specific amplification attempts of the *Oncorhynchus mykiss* oocyte protease inhibitor-1 gene, BAC RT492A1 (GenBank KC686349), failed to amplify a single locus, except within the BAC itself. Sequencing of PCR products of genomic DNA from the OSU female DH line yielded the cleanest reads, with primers F1/R1 showing discernible indels and SNPs and an overall homology of 94% as compared to the Y sequence, but it could not be determined whether this was a related sequence on the X chromosome or an autosomal homolog.

 Sex-linkage and SNP evaluations of the cAMP-responsive element-binding protein 3-like protein 4 (cr3l4) gene, BAC RT15B10 (GenBank KC686342) of DH RT genomic DNAs preferentially amplified paralogous loci; no primer combinations yielded sex-linked polymorphisms for this gene. 

Also within BAC RT15B10, the Zinc transporter ZIP1 (zip1) gene was evaluated by PCR and amplified product sequencing of RT clonal lines for sex-linked polymorphisms, which are referenced against the ZIP1 GenBank KC686350 deposition. The CREB-regulated transcription coactivator 2 (TORC2-like) gene was PCR evaluated for sex-linked polymorphisms in the RT clonal line panel, identifying 4 SNPs between the clonal lines, 1 sex-linked SNP, and a 17 bp deletion in SW only.

 Population and RT clone-specific polymorphisms were found in the DENN domain-containing protein 4B-like gene amplified in BAC RT15B10. All polymorphisms are referenced against the GenBank KC686345 sequence. RT15B10 also contains an interleukin enhancer-binding factor 2 pseudogene (GenBank KC686347); however the 2 primer sets used to confirm sequence within RT15B10 amplified more than one locus in all the DH genomic DNA analyzed.

In addition to these EST templates and annotated loci, a number of primers were designed to other unannotated regions within the Illumina/454 assembly contigs as well as to the homologous MMSRT draft genome scaffolds. Of these, there were 2 primer sets that yielded sex-specific SNPs and these are included in Table 3. 

## 4. Discussion

In this study we describe sequencing and CGH results from BAC clones forming a contig with the OmyY1 marker isolated by Brunelli and Wertzler [11] and the sdY sex determining gene isolated by Yano et al. [16]. Primers for the OmyY1 marker and the sdY gene amplified products from two of the BACs on the 3′ end of the contig. These BACs hybridized to the end of the short arm of the Y chromosome with some signal also on the end of the short arm of the X chromosome. Illumina sequencing of the BACs, because of the large number of repetitive sequences present, produced many small contigs. A number of putative genes were identified, and all genes PCR amplified appear to be present in both males and females, with only a small portion showing sex-specific sequence differences. Large contiguous sequences with over 95% homology to each other were found in nonoverlapping BACs suggesting that duplications of a number of regions, some of which containing putative genes, are present in contig #6256. Taken together these results suggest that the X and Y are in an early stage of differentiation, perhaps because infrequent episodes of crossing over have partially homogenized the content of the X and Y. 

 Because the rainbow trout X and Y chromosomes are usually morphologically distinct, it has been assumed that they are in a later stage of differentiation compared to species such as medaka where the X and Y are not distinguishable. In the case of medaka, the only difference between the X and Y is the insertion of the sex-determining gene DMY into the Y chromosome by transposition [32]. DMY was found in only one BAC clone. We analyzed a contig composed of a minimum tiling path of six BACs from the Swanson YY library. Sex-specific SNPs were identified in genes from BACs in the 5′ and middle of the contig, but few genes were found on the sdY on the 3′ end other than rDNA and transposable elements. Because the ends of contig #6256 contain very repetitive sequences, we were not able to extend the contig in either direction. It is possible that Y-specific genes are found in regions outside of the region that was sequenced. Future work could involve isolation of BACs and sequencing of these adjacent regions in both directions. 

The expression of all of the genes in the OmyY1 region could be examined to determine if any are expressed preferentially in males during sex determination or sexual differentiation. Isolation of the corresponding region from the OSU BAC library prepared from an XX female clonal line and comparison of the content and expression of genes on the X and the Y in the region adjacent to the proposed sex determination gene, sdY would provide information about the degree of divergence between the X and Y chromosomes. 

In the Swanson YY clonal line, the Y chromosome is not morphologically distinct from the X, with rDNA on the short arm adjacent to the centromere. This undifferentiated Y chromosome is possibly the result of crossing over between the X and Y in the population that gave rise to this clonal line. Future work on the male-specific region of the Y chromosome in YY clonal lines with the morphologically differentiated Y chromosome should reveal whether greater variation is present in the coding regions of the Y chromosome in such rainbow trout strains.

Finally, we would like to compare the male-specific content of the Y chromosome in the other *Oncorhynchus* species and determine the size of the region that is transposing to form new Y chromosomes in this genus. A BAC clone has been isolated that contains the sdY gene from a male Chinook salmon (RH Devlin, Fisheries and Oceans, pers. com.) and it will be interesting to compare the sequences of the adjacent regions in the two species. We are currently performing CGH experiments to compare the genes present in YY and XX genomic DNA from rainbow trout, coho salmon, and sockeye salmon which should also provide information on differences in gene content between the X and Y chromosomes of these species. 

## 5. Conclusions

We analyzed sequence data from a contig of rainbow trout BAC clones from the Swanson YY clonal line that includes the male-specific OmyY1 genetic marker and the sex-determining gene, sdY. Our data shows that this region is rich with rDNA and repetitive elements, but also has many predicted protein coding genes. Most genes in the region seem to be present on both the X and Y chromosomes, although sex-specific SNPs are found in a small portion of them, suggesting that the X and Y are at an early stage of differentiation. None of these genes were found in the cosmid or BAC clones from the Y chromosome of Chinook salmon (Phillips and Devlin, unpublished) and the two BAC clones containing OmyY1 and sdY did not hybridize to the Y chromosome of Chinook salmon, suggesting that the shared region containing the sex-determining gene between these two species is very small. 

## Supplementary Material

List of salmonid 4x44K oligo microarray hits from CGH analysis that share homology with the sequence from the OmyY1 region. This list includes single copy genes, paralogs to OmyY1 linked sequence, rDNA, transposon-related transcripts, and other repetitive elements.

## Figures and Tables

**Table 1:** List of predicted protein coding genes, pseudogenes, and unknown transcripts found in rainbow trout physical map contig #6256 sequence assemblies. Pseudogenes were differentiated from protein coding genes based on the presence of premature stop codons in open reading frames. The BAC clone column denotes the clone in the contig that gave a PCR product for the gene in question.

Gene name	Predicted gene function	PCR amplified in BAC clone	Annotation
Oncorhynchus mykiss sdY	Protein coding	143K08/223F06	Genbank AB626896.1
DENN domain-containing protein 4B-like (dennd4b-like)	Protein coding	RT015B10	Genbank KC686345
Immunoglobulin superfamily DCC subclass member 3-like (Igdcc3-like) [partial]	Protein coding	RT015B11	Genbank KC686344
cAMP-responsive element-binding protein 3-like protein 4 (cr3l4)	Protein coding	RT015B12	Genbank KC686342
F-box/WD repeat-containing protein 2 (FBXW2)	Protein coding	RT423A24	Genbank KC686346
Na/K/2Cl co-transporter (nkcc1a)	Protein coding	RT450D22	Genbank KC686348
Oocyte protease inhibitor-1 (opi-1)	Protein coding	RT004L12	Genbank KC686349
Zinc/iron-regulated protein (zip1)	Protein coding	RT004L12	Genbank KC686350
CREB-regulated transcription coactivator 2-like (TORC2-like)	Protein coding		Genbank KC686350
Zinc finger CCHC domain-containing protein 3-like	Protein coding		Genbank KC686343
General transcription factor II-I repeat domain-containing protein 2-like	Protein coding		Genbank KC686351
rRNA promoter binding protein	Protein coding		Genbank KC686343
Interleukin enhancer-binding factor 2 (ILF-2)	Pseudogene	RT015B10	Genbank KC686347
Unknown (Some homology to laminin subunit beta-1-like)	Unknown		cGRASP EST omyk-BX860091
Unknown	Unknown		cGRASP EST omyk-CB488087
Unknown (Some homology to Sec16A)	Unknown		cGRASP EST Contig22207
Unknown	Unknown		cGRASP EST omyk-DV199273
Unknown	Unknown		cGRASP EST Contig19333

**Table 2:** List of predicted nonrepetitive transcribed genes in rainbow trout physical map contig #6256 with corresponding EST's in at least one database. EST designations in bold had the highest homology to OmyY1 linked sequence, and those sequences were used for homology and coverage analyses.

Gene name	4 × 44 K salmonid oligo array designation	cGRASP EST cluster contig annotation	GenBank Annotation	Miller draft rainbow trout genome scaffold	Miller scaffold homology (base pair identities)	Coverage in Miller scaffold	OmyY1 contig (Illumina unless noted)	OmyY1 contig homology (base pair identities)	Coverage within OmyY1 contig	PCR amplification in BAC clone	Transcribed gene in OmyY1 contig?
Oncorhynchus mykiss sdY			**AB626896.1**	n/a	n/a	n/a	454 contig_3	3270/3273	100%	143K08/223F06	Yes
F-Box/WD repeat containing protein 2 (FBXW2)	C262R066/ C179R024	**Contig53026**		MMSRT112A/ 121C (overlap)	2319/2326	100%	Contig_2	2319/2326	100%	RT450D22/ RT492A21	Yes
Unknown (Some homology to laminin subunit beta-1-like)		**omyk-BX860091**		MMSRT112A/ 121C (overlap)	747/778	100%	Contig_2	747/778	100%		Likely
Na/K/2Cl co-transporter (nkcc1a)	C029R137	**omyk-CR375193**		MMSRT112A	832/843	100%	Contig_2	832/843	100%	RT450D22	Yes
Oocyte protease inhibitor-1 (opi-1)		**Contig59765**		MMSRT010A/ 121C	919/940, 883/906	97%, 94%	Contig_2	883/906	94%		Likely in MMSRT010A scaffold
Unknown		**omyk-CB488087**		MMSRT010A/ 121C	315/315, 311/315	100%, 100%	Contig_2	311/315	100%		Likely in MMSRT010A scaffold
Unknown (Some homology to Sec16A)		**Contig22207**		MMSRT104C	1193/1197	100%	Contig_28	1193/1197	100%		Yes
cAMP-responsive element-binding protein 3-like protein 4 (cr3l4)	C264R086	**Contig51133**		MMSRT014G	1173/1181	99%	Contig_121	1173/1181	99%	RT15B10	Yes
CREB-regulated transcription coactivator 2 (TORC2)	**C117R024**			MMSRT069D*	657/726	97%	Contig_107	698/748	100%	RT15B10	Possibly
Zinc/iron-regulated protein (zip1) (splice A)	**C053R110**			MMSRT069D*	391/415	39%	Contig_108, 110	1058/1065	100%	RT15B10	Yes
Zinc/iron-regulated protein (zip1) (splice B)	C014R029	**CLUSTER_ID#5374769**		MMSRT069D*	916/1137	99%	Contig_108	1125/1148	100%	RT15B10	Likely
Unknown		**omyk-DV199273**		MMSRT069D*	451/518	74%	Contig_135, 153	696/700	100%		Yes
Unknown		**Contig19333**		MMSRT069D*	637/755	87%	Contig_119	815/816	94%		Yes

*Denotes scaffolds from Michael Miller's draft genome that are paralogous to OmyY1 linked sequence.

**Table 3:** Primer information for markers in the OmyY1 BAC contig (#6256).The parental lines are the doubled haploid male (AR/SW) and female (OSU/WR) rainbow trout clonal lines in which the products were amplified. Clonal lines listed on either side of “/” denote which clonal lines have the SNP or indel. The last column shows the base pair location of the SNP(s) or indel in the amplified product.

Mapping primers	Gene	Primer name	Tm	BAC clone	Product size	Parental line	SNP or indel
F1:GCCTATCAACCTCCTGTACTT R1:CCTGCGAAATAACCAATG	Fbxw2	Fbxw2-F/R	58–60	450D22/492A1	703 bp	AR, SW/OSU, WR	261bp
F2:CTACAGGAAGTCCAAACGAGG R2:TGGGGATGGTAGTTGACAGTC	Fbxw2	Fbxw2-F2/R2	58–64	450D22/492A21	740 bp	AR, SW/OSU, WR	162(A/G) 525(T/A)
F3:TAGACATTTCCCCTGTATTACC R3:AAAAGGGTCTGCTACTCATT	Fbxw2	Fbxw2-F3/R3	58	450D22/492A21	818 bp	AR, SW/OSU, WR	378(G/T), 530(G/C) 756(G/A), 642–46 bp
F4:CATGTCTTCTTTCCATTGGCC R4:TTGTGACCTCCATCTTGTAGG	Intergenic	c451-grp2-F/R	62	450D22	392 bp	AR, SW/OSU, WR	147(G/T), 166(G/T)
F5:CGACAGCACCAAACTAATCTTT R5:GATCATCAGTTGCACGGAGG	Intergenic	c35-450D22-F2-R2	62	450D22	978 bp	AR, SW/OSU, WR	male-specific amplification
F6:TTACAGTGACATTCGGTTCAA R6:TTTGGGTGTGTGATGCTATTT	Intergenic	MM121C-5′14kb	62	450D22/492A21	687 bp	AR/SW/OSU/WR	168(G/A)
F7:CGGAATGCACCAAACCCTAA R7:GTATGTTGTCCTTGGCTCCGAA	Intergenic	1M42E-14201- F5/14944-R	65	423A24	743 bp	AR/OSU	AFLP in AR
